# The gut microbiota modulates responses to anti–PD-1 and chemotherapy combination therapy and related adverse events in patients with advanced solid tumors

**DOI:** 10.3389/fonc.2022.887383

**Published:** 2022-10-25

**Authors:** Zhaozhen Wu, Sujie Zhang, Lingling Li, Ziwei Huang, Di Huang, Yi Hu

**Affiliations:** ^1^ Department of Medical Oncology, the Fifth Medicine Center of Chinese People’s Liberation Army (PLA) General Hospital, Beijing, China; ^2^ Beijing Chest Hospital, Beijing, China; ^3^ School of Medicine, Nankai University, Tianjin, China

**Keywords:** gut microbiota, response, adverse events, anti–PD-1 therapy, solid tumors

## Abstract

**Background:**

Immune checkpoint inhibitors (ICIs) targeting programmed cell death protein 1 (PD-1) have been widely used in treating different malignancies. Several studies have reported that the gut microbiota modulates the response and adverse events (AEs) to ICIs in melanoma, non–small cell lung cancer (NSCLC), renal cell cancer and hepatocellular carcinoma, but data on other cancer types and ICI combination therapy are limited.

**Methods:**

Stool samples were collected from patients with cancer who received anti–PD-1 and chemotherapy combination treatment and were analyzed by fecal metagenomic sequencing. The microbiota diversity and composition were compared between the responder (R) and non-responder (NR) groups and the AE vs. the non-AE (NAE) groups. In addition, associated functional genes and metabolic pathways were identified.

**Results:**

At baseline, the microbiota diversity of the groups was similar, but the genera *Parabacteroides*, *Clostridia bacterium UC5.1_2F7*, and *Bifidobacterium dentium* were enriched in the R group, whereas *Bacteroides dorei* and 11 species of *Nocardia* were enriched in the NR group. At 6 weeks, the beta diversity was significantly different between the R and NR groups. Further analysis found that 35 genera, such as *Alipes*, *Parabacteroides*, *Phascolarctobacterium*, *Collinsella*, *Ruminiclostridium*, *Porphyromonas*, and *Butyricimonas* and several genera of the *Fibrobacteraceae* family, were frequently distributed in the R group, whereas 17 genera, including *Enterococcus*, *Lachnoclostridium*, *Hungatella*, and *Bilophila* and several genera of the *Pseudonocardiaceae* and *Beijerinckiaceae* families, were more abundant in the NR group. A total of 66 and 52 Kyoto Encyclopedia of Genes and Genomes (KEGG) orthologs (KOs) were significantly enriched in the R and NR groups, respectively. In addition, pathway analysis revealed functional differences in the gut microbacteria in the R group, including the enrichment of anabolic pathways and DNA damage repair (DDR) pathways. Dynamic comparisons of the bacterial composition at baseline, 6 weeks, and 12 weeks showed that the abundance of *Weissella* significantly increased in the R group at 6 weeks and the abundance of *Fusobacterium* and *Anaerotruncus* significantly increased in the NR group at 12 weeks. Linear discriminant analysis effect size analysis indicated that bacteria of *Bacteroidetes*, especially *Bacteroides*, were enriched in the NAE group, whereas flora of *Firmcutes*, such as *Faecalibacterium prausnitzii*, *Bacteroides fragilis*, and *Ruminococcus lactaris*, were enriched in the AE group.

**Conclusion:**

Beta diversity and differences in the gut microbiota modulated AEs and the response to anti–PD-1 blockade combined with chemotherapy, by regulating related anabolic and DDR pathways. Dynamic changes in the intestinal microbiome may predict the efficacy of PD-1 inhibitor–based therapy.

## Introduction

In recent years, immune checkpoint inhibitors (ICIs), which target the cytotoxic T lymphocyte antigen-4 (CTLA-4) or programmed cell death protein 1 (PD-1)/PD-1 ligand (PD-L1) pathway, have been shown to be effective in the treatment of different malignancies (1) with a lower incidence of side effects. However, only a subset of patients with cancer derives benefits from these agents. Several biomarkers, such as PD-L1, tumor mutation burden and other molecular characteristics, microsatellite instability, and Epstein–Barr virus (2–7), were suggested to help select potential beneficiaries, but none of them was accurate. It is thus critical to further understand the determinants driving response and explore potential biomarkers.

Increasing evidence suggests that the gut microbiome plays a significant role in the response to both immunotherapy and chemotherapy. Significantly, it has been reported that the diversity and composition of the intestinal microbiota influence ICI responses in both mouse models and patients. In murine models, it was demonstrated that *B. thetaiotaomicron* and *B. fragilis* were associated with the response to CTLA-4 blockade (8), and *Bifidobacterium* was associated with the response to PD-L1 blockade (9, 10). *Clostridiales* and *Bacteroidales* were recently reported to be associated with favorable and unfavorable responses, respectively, to anti–PD-1 immunotherapy in a cohort of 112 patients with melanoma (11). It was reported that an elevation of *Prevotella/Bacteroides* ratio was related with a favorable response to anti–PD-1 therapy in advanced-stage gastrointestinal cancer (12). In addition, *Ruminococcaceae* was found to be associated with a favorable response to ICI in NSCLC (13). Moreover, *Akkermansia muciniphila* was found to enhance the efficacy of PD-1 blockade in epithelial tumors in an interleukin-12 (IL-12)–dependent manner (14, 15). In addition to the influence of the gut microbiome on the response to immunotherapy, several studies have reported the efficacy of chemotherapeutic drugs, such as cyclophosphamide and platinum, to be modulated by gut microbiota (16, 17). This is important because combination therapies with anti–PD-1/PD-L1 and other drugs, such as chemotherapy and anti-angiogenesis drugs, are more commonly used than single- agent ICIs in real-word clinical settings. Therefore, we aimed to explore the effect of the gut microbiota on PD-1 inhibitor–based combined therapy.

## Method

### Study design and fecal sample collection

In total, 27 patients with cancer treated with anti–PD-1 combined with chemotherapy every 3 weeks in the Oncology Department of Chinese PLA General Hospital were enrolled in this study. No antibiotics were used during this treatment regimen, and all patients had signed informed consent forms and donated their stool samples for this study. Stool samples from 24 patients at baseline (day 0), 6 weeks, and 12 weeks were collected and immediately stored at −80°C until DNA extraction was performed, whereas the stool samples from three patients with AEs were collected only at baseline. The gut microbiota was analyzed by metagenomic sequencing. Clinicopathological data and treatment information were independently collected and recorded by two physicians, and all patients were classified as responders (R, complete response, partial response, or stable disease ≥ 6 months; n = 16) and non-responders (NR; disease progression or stable disease < 6 months; n = 8) based on radiological evaluation by two radiologists according to the Response Evaluation Criteria in Solid Tumors, version 1.1 (RECIST 1.1). Patients were classified as the AE group and the non-AE (NAE) group according to the National Cancer Institute Common Terminology Criteria for Adverse Events, version 4.0 (CTCAE 4.0). This study was approved by the Institutional Ethics Committee of Chinese PLA General Hospital, Beijing, China (approval number: S2019-184-01).

### DNA extraction and metagenomic sequencing

The gut bacterial composition was evaluated by fecal metagenomic sequencing. Briefly, bacterial genomic DNA was extracted using the QIAamp DNA Stool Mini Kit (Qiagen, Hilden, Germany). After the integrity and concentration of DNA was determined, individual libraries were constructed with the NEBNext Ultra DNA Library Prep Kit for Illumina (BGI, Shenzhen, China), loaded onto the BGISEQ-500 RS platform (BGI, Shenzhen, China), and then were sequenced according to a 2 × 100-bp paired-end read protocol. Quality filtering, trimming, and demultiplexing were performed as described previously (18). A total of 70 datasets were generated. Overall, 85.84% of the raw reads were considered as high quality, with an average length of 150 bp and an average Q30 score of 88.54% (**eTable 1**). The species accumulation curves are shown in **eFigure 1**.

### Taxonomic and gene profiling

All high-quality reads were aligned to the Homo sapiens (human) genome assembly hg38 using SOAPalign 2.21 (https://anaconda.org/bioconda/soapaligner) with default parameters to remove human reads. The retained clean reads were aligned to ~1 M clade-specific marker genes from approximately 17,000 reference genomes to estimate relative phylotype abundance using MetaPhlAn (version 2.5.0). For gene annotation, the clean reads were aligned to the integrated gene catalog (IGC) by using SOAPalign 2.21 with the default parameters; only the reads with both ends mapped to the same gene were used in the subsequent analysis. An average IGC mapping rate of 53% was achieved. Functional annotations were carried out *via* BLASTP searches against the Kyoto Encyclopedia of Genes and Genomes (KEGG) database (e-value ≤1e^− 5^ and high-scoring segment pair scoring >60). The KEGG ortholog (KO) abundance was estimated by accumulating the relative abundance of all genes belonging to this feature. All of the above procedures were carried out as previously described (19).

### Statistical analysis

Clinical characteristics and demographic data were summarized *via* descriptive statistical analysis, and the Fisher’s exact test was used to compare differences between two groups using SPSS 20.0 software (IBM, SPSS, Chicago, IL, USA). Two-sided P-values were evaluated, and P < 0.05 was considered statistically significant. The Shannon and Simpson indices were used to calculate alpha diversity, and principal coordinates analysis (PCoA) and the analysis of similarities (ANOSIM) test with the Bray–Curtis distance were used to evaluate beta diversity. A non-parametric Wilcoxon rank sum test was applied to analyze the statistical significance of diversity indices, taxa, and KOs between the R and NR groups. The linear discriminant analysis (LDA) effect size (LEfSe) algorithm was further used to identify the phylotypes that had a significantly different abundance in different groups; phylotypes with an LDA score cutoff of 2.0 and P < 0.05 in built-in rank sum test were regarded as statistically significant. A ternary plot in the R language (ggtern package) was used to compare differences among three groups. Spearman sequential correlation analysis was used to estimate the correlation between two variables (microbiota family and metabolic pathways in KEGG database, by R language), and only significant correlations with P < 0.01 and rho > 0.5 are shown. The network was visualized with Cytoscape 3.0.2.

## Results

### Patient characteristics

In total, 27 patients with cancer (including three patients without serial stool samples) were enrolled in our study, including 12 patients with non–small cell lung cancer (NSCLC), nine patients with digestive system cancers, and one patient each with ovarian, bladder, and prostate cancer, separately. We collected patients’ demographics and clinicopathological data, including baseline antibiotic use (within 1 month), metastasis, number of treatment regimens received, PD-L1 expression, tumor mutation burden, lymphocyte number, and Lactate dehydrogenase (LDH). We placed these patients in the R or NR group, and the clinical characteristics were balanced between these groups (**Table 1**).

**Table 1:** Clinical data of patients in the R and NR groups.

Characteristic	R group (n = 16)	NR group (n = 8)	P value
Gender			1.000
Male	10	5	
Female	6	3	
age			0.362
<65	13	5	
≥65	3	3	
ECOG			0.249
0–1	15	6	
≥2	1	2	
Smoking history			0.388
Current or former	8	2	
Never	8	6	
Antibiotic use			1.000
In ≤1month	3	2	
No	13	6	
Metastasis number			1.000
number<2	12	6	
number≥2	4	2	
CNS metastasis			0.536
yes	2	0	
no	14	8	
Liver metastasis			0.065
yes	2	0	
no	14	8	
Lung metastasis			0.621
yes	5	1	
no	11	7	
Bone metastasis			0.363
yes	4	4	
no	12	4	
PD-L1 expression			0.191
<1%	3	4	
≥1%	12	3	
unknown	1	1	
TMB			0.095
<10m/Mb	5	6	
≥10m/Mb	6	0	
unknown	5	2	
Lymphocyte number			0.167
<0.8*10^9^/L	3	4	
≥0.8*10^9^/L	13	4	
LDH			1.000
<250U/L	11	5	
≥250U/L	5	3	
Treatment lines			0.741
1	7	5	
1~2	5	2	
≥3	4	1	

### Dominant microbiota composition and diversity

**Figure 1:**
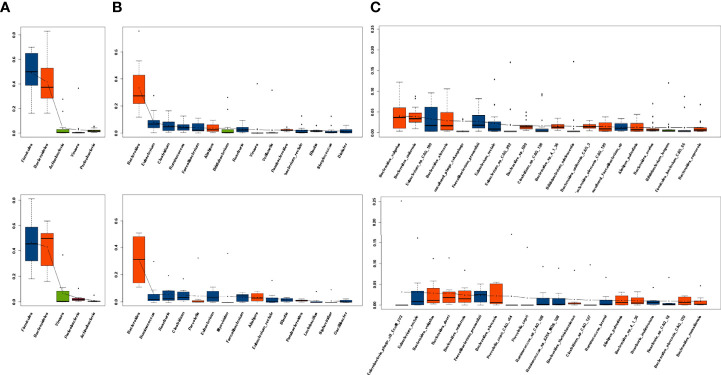
Dominant microbiota composition at baseline. **(A)** The top 5 phyla, **(B)** top 10 genera, and **(C)** top 20 species in the R group (n = 16, upper) and NR group (n = 8, lower) are listed separately. Red indicates *Bacteroidetes* and blue indicates *Firmicutes*.

The top five phyla, top 10 genera, and top 20 species in the R group and the NR group at baseline are listed separately. At baseline, Gram-positive *Firmicutes* and Gram-negative *Bacteroidetes* dominated the fecal microbiota of both the R and NR groups at the phylum level, which was in accordance with the previous findings in healthy human (20), followed by *Actinobacteria*, *Viruses*, and *Proteobacteria* in the R group and *Viruses*, *Proteobacteria*, and *Actinobacteria* in the NR group (**Figure 1A**). At the genus level, the dominant microbiome at baseline was similar between the two groups, except for *Alistipes* and *Bifidobacterium*, which seemed to be more abundant in the R group, and *Myoviridae* and *Siphoviridae*, which seemed to be more dominant in the NR group (**Figure 1B**). At the species level, the top 20 species of two groups almost all belonged to *Firmicutes* and *Bacteroidetes* (**Figure 1C**). The microbiome composition of 24 patients at baseline is shown in **eFigure 3A**.

The dynamic analysis of the dominant microbiota at the phylum, genus, and species levels showed a significant decrease in *uncultured phage crAssphage* at 12 weeks compared with baseline in the NR group (*P =* 0.047; **eFigure 2F**); however, no other significant differences at baseline, 6 weeks, and 12 weeks were observed in either the R group (**eFigures 2A, C, E**) or the NR group (**eFigures 2D, F**, **3B**).

**Figure 2:**
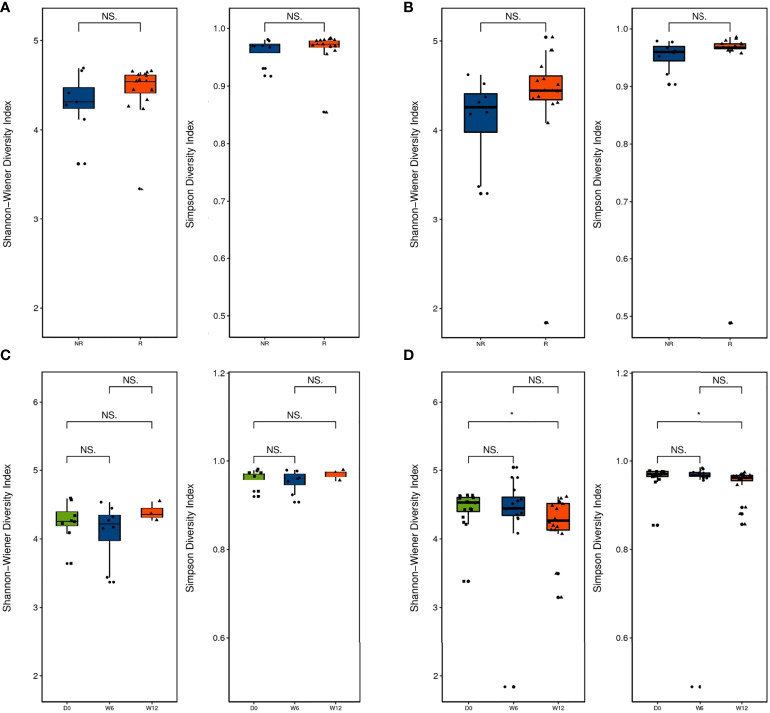
Diversity of the gut microbiota between the R group (n = 16) and NR group (n = 8, except at 12 weeks). **(A)** Alpha diversity was evaluated by the Shannon and Simpson indices at baseline and **(B)** 6 weeks. **(C)** Comparison of alpha diversity by the Shannon and Simpson indices at baseline, 6 weeks, and 12 weeks in the R group and **(D)** the NR group (n = 3 at 12 weeks). NS and *indicate values of P > 0.05, P < 0.05, respectively.

**Figure 3:**
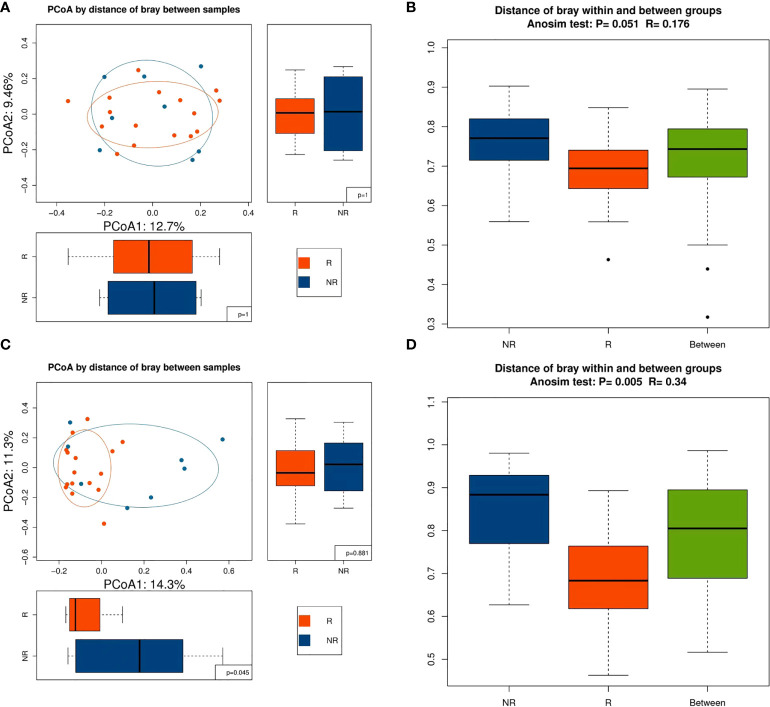
Beta diversity between the R (n = 16) and NR (n = 8) groups. **(A)** Beta diversity was evaluated by PCoA and **(B)** ANOSIM at baseline. **(C)** Beta diversity was evaluated by PCoA and **(D)** ANOSIM at 6 weeks.

Alpha diversity was higher in the R group than in the NR group at baseline and 6 weeks, but the differences were not significant (*P >* 0.05; **Figures 2A, B**). Alpha diversity was higher at 12 weeks than at baseline in the R group, but a significant difference was not observed (*P >* 0.05; **Figure 2C**), whereas alpha diversity significantly decreased at 12 weeks compared with baseline in the NR group (0.01 < *P* < 0.05; **Figure 2D**). Although beta diversity determined by PCoA and ANOSIM indicated no significant difference at baseline between the R group and the NR group (*P >* 0.05; **Figures 3A, B**), beta diversity was significantly different by both PCoA (*P*_1_ = 0.045; **Figure 3C**) and ANOSIM (*P =* 0.005, *R* = 0.34; **Figure 3D**) at 6 weeks. These results suggest that the intragroup diversity (measured by alpha diversity) was similar at baseline and 6 weeks in the two groups but significantly decreased at 12 weeks in the NR group. In addition, the intergroup diversity (indicated by beta diversity) was significantly different between the R and NR groups at 6 weeks.

### Comparison of the gut microbiota composition and genes between the two groups

**Figure 4:**
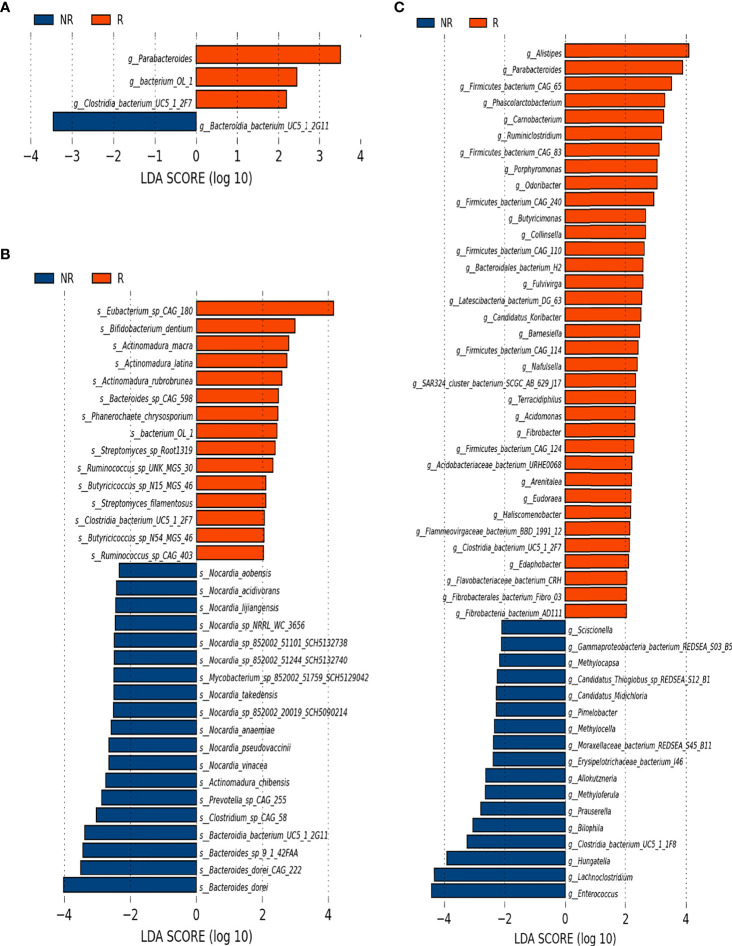
Microbiota composition differences between the R group (n = 16) and the NR group (n = 8). **(A)** The relative abundance of R-enriched and NR-enriched genera and **(B)** species at baseline. **(C)** The relative abundance of differentially abundant genera at 6 weeks, as identified by LEfSe analysis (Kruskal–Wallis sum rank test, P < 0.05).

To further investigate whether the composition of the gut microbiota influenced the response to anti–PD-1–based combination therapy, we compared the relative abundance of gut microbiome components at the genus and species levels in further detail between the R group and the NR group. At baseline, LEfSe analysis of the two groups indicated that the genera *Parabacteroides*, *Bacterium OL_1* and *Clostridia bacterium UC5.1_2F7* genus, and 15 species, including *Bifidobacterium dentium*, were enriched in the R group, whereas the genus *Bacteroidia bacterium UC5.1_2G11* and 19 species including *Bacteroides dorei* and 11 species of *Nocardia* genus were enriched in the NR group (**Figures 4A, B**). At 6 weeks, differences in the microbiome were more noticeable. A total of 35 genera, such as *Alipes*, *Parabacteroides*, *Phascolarctobacterium*, *Collinsella*, *Ruminiclostridium*, *Porphyromonas*, *Butyricimonas*, and several genera of *Fibrobacteraceae* family, were frequently founed in the R group; whereas 17 genera, including *Enterococcus*, *Lachnoclostridium*, *Hungatella*, *Bilophila*, several genera of *Pseudonocardiaceae*, and *Beijerinckiaceae* families, were more abundant in the NR group (**Figure 4C**). At the species level, 66 species in the R group and 17 species in the NR group were identified as being differentially abundant by LEfSe analysis. Eubacterium siraeum, Bacteroides uniformis, *Bacteroides xylanisolvens*, *Bacteroides salyersiae*, *Bacteroides caccae CAG_21*, *Bacteroides fragilis*, *Alistipes putredinis*, *Parabacteroides merdae*, *Ruminococcus bromii*, *Lactobacillus kitasatonis*, *Collinsella aerofacien*, and many short-chain fatty acid (SCFA)–producing species were included in the R group, and *Hungatella hathewayi*, several species of the genus *Enterococcus* genus, *Erysipelotrichaceae bacterium I46*, *Bilophila wadsworthia*, and *Lactobacillus phage A2* were included in the NR group (**eFigure 3B**). The difference in the composition of the gut microbiome at 12 weeks could not be quantified because five samples in the NR group were not obtained, which resulted in an imbalance in case numbers.

**Figure 5:**
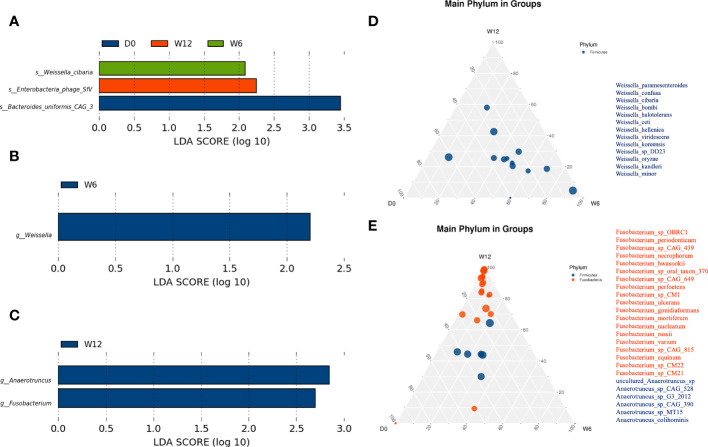
Dynamic analysis of the gut microbiota longitudinally. **(A)** Differential species in the R group (n = 16) at baseline, 6 weeks, and 12 weeks. **(B, C)** Differential genera in the R group (n = 16) and the NR group (n = 8 at 6 weeks, n = 3 at 12 weeks). **(D, E)** At the genus level, significantly different species were identified by ternary plots between the R group (n = 16) and NR group (n = 8) at 6 weeks.

We further compared longitudinal differences in the gut microbiota at baseline, 6 weeks, and 12 weeks. LEfSe analysis identified three species in the R group: *Bacteroides uniformis CAG_3* (D0), *Weissella cibaria* (W6), and Enterobacteria phage SfV(W12) (**Figure 5A**). At the genus level, *Weissella* was significantly enriched at 6 weeks in the R group (**Figure 5B**), whereas *Fusobacterium* and *Anaerotruncus* were significantly enriched at 12 weeks in the NR group (**Figure 5C**). Ternary plots generated at the species level yielded similar results to LEfSe analysis (data not shown), and ternary plots generated at the genus level showed the differential enrichment of species of the genera *Weissella, Fusobacterium*, and *Anaerotruncus genus* (**Figures 5D, E**).

At 6 weeks, ANOSIM showed a significant difference in genes related to the microbiome between the two groups (*P =* 0.004, *R* = 0.347). Coabundance genes (CAG) analysis found 65 differential genes at different taxonomic levels (**eTable 2**). Some distinct microbiota components between the two groups also showed significant differences at the genetic level; these included *Alipes*, *Parabacteroides merdae*, *Eubacterium siraeum*, *Bacteroides caccae*, *Lactobacillus salivarius*, *Bacteroides fragilis*, and *Barnesiella intestinihominis YIT 11860* in the R group and *Bilophila* and *Bilophila wadsworthia* in the NR group (**eTable 2**).

### Analysis of functional genes and enriched pathway

**Figure 6:**
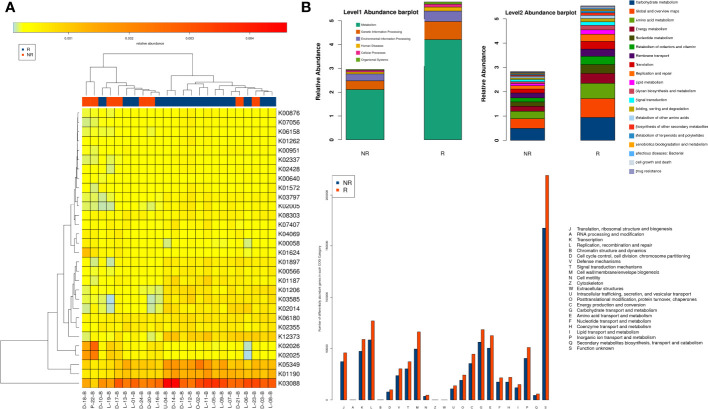
Comparison of functional genes and metabolic pathways at 6 weeks in the R group (n = 16) and the NR group (n = 8). **(A)** Heatmap of samples and differential KOs based on LEfSe analysis. **(B)** The gut microbiota of the R group was more enriched in biochemical and metabolic pathways at different functional levels, as evaluated with the KEGG and EggNOG databases.

Functional gene families associated with bacteria in the two groups were further investigated. ANOSIM showed a significant intergroup difference in KEGG orthologs only at 6 weeks (*P =* 0.004, *R* = 0.335). A total of 66 and 52 KOs with significant differences were more enriched in the R and NR groups, respectively (**eTable 3**), and a heatmap of samples and distinct KOs is shown (**Figure 6A**). Analysis at different functional levels all indicated that the gut microbiota of the R group was more enriched in biochemical functions than that of the NR group (**Figure 6B**). Further correlation analysis of differentially abundant microbiota and KEGG pathways revealed functional differences in the gut microbiota in the R group, including the enrichment of anabolic pathways and DNA damage repair (DDR) pathways, such as amino acid metabolism, the biosynthesis of other secondary metabolites, and cancer: overview, energy metabolism, glycan biosynthesis and metabolism, part of lipid metabolism, and transcription and translation at both the genus level (**eFigure 4**) and species level (**eFigure 5**).

### Correlation of the gut microbiota and immune-related adverse events

On the basis of the presence or absence of AEs, seven patients were placed in the AE group and 20 patients were in the NAE group, and data from these 27 patients were analyzed. All immune-related adverse events (irAEs) were ≥ grade 2, including five cases of pneumonitis and two patients of immunotherapy-induced colitis. With the exception of one patient with grade 2 pneumonitis, who restarted immunotherapy after pneumonitis was well-controlled, patients with irAEs discontinued immunotherapy.

**Figure 7:**
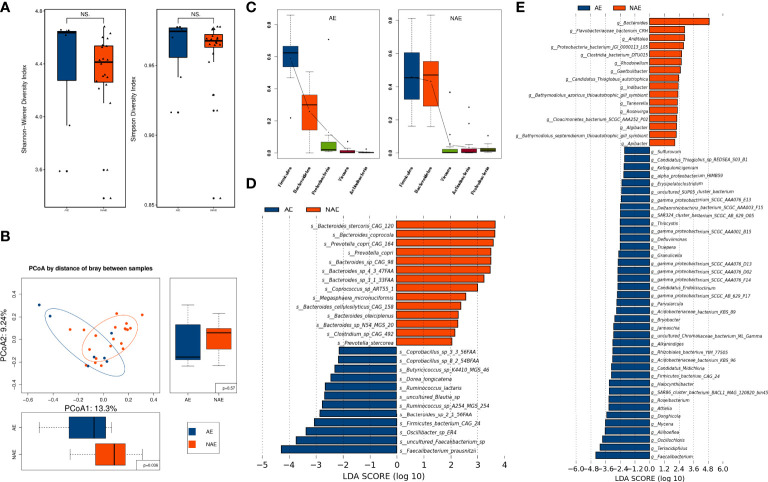
The correlation of the baseline gut microbiome and irAEs. **(A)** Alpha diversity was determined by the Shannon and Simpson indices at the species level between the AE group (n = 7) and the NAE group (n = 20). **(B)** Beta diversity was evaluated by PCoA analysis at the species level. **(C)** Dominant gut microbiota at the phylum level. **(D)** Differentially abundant microbiota components were identified by LEfSe analysis at the genus and **(E)** species levels.

Although alpha diversity (at the species level) showed no significant difference between the AE group and NAE group (*P >* 0.05; **Figure 7A**), PCoA of beta diversity indicated that there was a significant intergroup difference between these two groups (*P =* 0.036; **Figure 7B**). At the phylum level, *Firmicutes* and *Proteobacteria* were more abundant in the AE group, and *Bacteroidetes* was more abundant in the NAE group (**Figure 7C**). At the genus level, LEfSe analysis identified 38 AE-enriched genera and 16 NAE-enriched genera, including many genera from the *Bacteroides* (**Figure 7D**). At the species level, LEfSe analysis indicated that 12 species (most of them belonging to *Firmicutes*) were correlated with non-AEs, whereas 13 species of *Firmcutes*, such as *Faecalibacterium prausnitzii*, and *Bacteroides* sp. *2_1_56FAA* (*Bacteroides fragilis*) were enriched in the AE group (**Figure 7E**).

The results of LEfSe analysis revealed that 12 and 48 KOs were significantly different in the AE and NAE groups, and distinct KOs within the groups are listed in the heatmap (**eFigure 6A**). FishTaco analysis suggested that NAE-enriched species of *Bacteroides* promoted type I polyketide biosynthesis (**eFigure 6B**). Further investigation found that irAEs were positively correlated with energy metabolism, membrane transport, transcription, and translation and that irAEs were negatively correlated with streptomycin metabolism, penicillin and cephalosporin biosynthesis, glycan biosynthesis and metabolism, sphingolipid metabolism, and steroid hormone biosynthesis (**eFigure 7**).

## Discussion

Recent work has highlighted the key role of the gut microbiota in mediating tumor responses to chemotherapeutic agents and ICIs as well as irAEs (15, 21, 22). Akkermansia muciniphila (Akk) has been reported to be associated with clinical benefit of ICI in patients with NSCLC or kidney cancer (14, 23), and intestinal Akk was reported to be accompanied by a richer commensalism, including *Bifidobacterium adolescentis* and *Eubacterium hallii (* 15). The diversity of gut microbiota was reported to influence the response to ICIs (19, 24), and in our study, the Shannon and Simpson indices were higher in the R group than in the NR group, but a significant difference was not reached, perhaps due to the small case number. Longitudinal comparisons showed that alpha diversity increased in the R group and decreased in the NR group at 12 weeks. Our study also showed that beta diversity markedly influenced the efficacy of PD-1 blockade–based combination therapy. Baseline beta diversity evaluated by ANOSIM showed a critical P-value of 0.051 (**Figure 3B**), and beta diversity evaluated by PCoA was not significantly different at baseline between the R group and the NR group (*P*_1_ = 1, *P*_2_ = 1; **Figure 3A**); this might have been due to the strict standards of patient selection and sample collection. Beta diversity at 6 weeks by both two methods showed a significant difference between the R group and the NR group, and this result was consistent with a previous study that showed that the beta diversity became significantly lower as early as week 6 (19). It is worthy of further study on the relationship of beta diversity and the efficacy of immunotherapy at different timepoints.

We identified differentially enriched gut microbiota at both baseline and 6 weeks in the R and NR groups; *Parabacteroides* and *Bifidobacterium dentium* were more abundant in the R group at baseline, which was in accordance with previous studies (9, 25). The differential enrichment between the two groups was more notable at 6 weeks than at baseline; 35 genera and 66 species were enriched in the R group. Among these enriched microbiota components, *Alipes (* 14), *Bacteroides fragilis (* 8), Eubacterium siraeum (14), *Ruminococcus bromii (* 19), *Collinsella aerofacien* and *Parabacteroides merdae (* 25), *Bacteroides caccae CAG_21 (* 26), and *Bacteroides salyersiae* and *Bacteroides xylanisolvens (* 27) were previously reported to be favorable for antitumor responses to ICI. At the same time, we identified 17 genera and 17 species that were significantly enriched in the NR group, of which *Hungatella hathewayi* had been previously reported to induce resistance to ICIs in renal cell carcinoma (27). Analysis at the genetic level further confirmed that *Alistipes*, *Parabacteroides merdae*, and *Eubacterium* sp. *CAG_180* were enriched in the R group, and this consistency at the genus, species, and genetic levels supported the validity of our results.

Although previous investigations reported interesting results and conclusions, we found little congruence among the different studies in the specific bacteria that were found to be favorable for antitumor responses. Therefore, longitudinal detection may play an important role. In our study, the longitudinal analysis of the composition of gut microbiota showed that the abundance of *Weissella*, especially *Weissella cibaria*, increased in the R group, whereas the abundance of *Fusobacteria* increased in the NR group. *Weissella cibaria*, a kind of lactic acid bacteria, has been reported to have the potential to prevent cancer (28), and different studies have indicated that *Fusobacteria* modulates the tumor microenvironment, leading to colon cancer growth and poor outcomes (29, 30).

We also found that patients with a higher abundance of *Bacteroidetes* in the gut microbiota were more likely to be protected from irAEs (pneumonitis and colitis) in our study, especially *Bacteroides* at the genus level and *Faecalibacterium prausnitzii* at the species level. This might be explained by the fact that *Bacteroidetes* promoted the biosynthesis and metabolism of anti-inflammatory components, including streptomycin metabolism, penicillin and cephalosporin biosynthesis, glycan biosynthesis and metabolism, sphingolipid metabolism, and steroid hormone biosynthesis (**eFigure 8**).

Increasing evidence has shown that the mechanisms through which microbacteria promote ICI responses could involve promoting the biosynthesis of amino acids and SCFAs, which regulate the immune response by inhibiting histone deacetylase (31). Our results indicated that anabolic pathways, including amino acid and fatty acid biosynthesis and metabolism, were correlated with gut microbiota components enriched in the R group. In addition, we found that DDR pathways, including homologous recombination, mismatch repair, DNA replication, and nonhomologous end joining, were markedly related to gut microbiota components that were enriched in the R group. Chemotherapy induces additional DNA damage, and a high level of the DDR was correlated with better efficacy of ICIs (32), which might be one of the reasons why those microbiota promoted the response to ICI-based combination therapy.

Mechanistically, the microbiome has been reported to affect tumor-specific CD8^+^ T cells *via* TLR4 or TLR9/MyD88 signaling and the IL-12 pathway (33, 34). One study showed that *B. fragilis* facilitated the efficacy of CTLA-4 blockade *via* IL-12–induced Th1 response and dendritic cell maturation (8), and another study showed that *Akkermansia muciniphila* modulated the efficacy of PD-1 blockade in an IL-12–dependent manner by upregulating the recruitment of CCR9^+^CXCR3^+^CD4^+^ T cells to tumor beds (14). We examined PD-L1 expression and lymphocyte numbers, but correlation analysis with the gut microbiota showed no significant differences between the N and R groups. Therefore, the absence of further mechanistic exploration of cytokines such as IL-12 and interferon-γ and lymphocyte classification and function was a limitation of our study.

To our knowledge, this is the first study to report the relationship between gut microbiome and combined anti–PD-1 treatment/chemotherapy in cancer *via* the dynamic detection of the gut microbiome using metagenomic sequencing. Nevertheless, there are some limitations in our study. First, the drugs, cancer types, and treatment lines were not exactly the same between groups; therefore, a correlation analysis of the gut microbiome and survival could not be conducted. Second, immunophenotyping was not performed and used to analyze how the gut microbiota affected the tumor microenvironment. Last, fecal bacteria transplantation and gavage administration of probiotics were not carried out, as this was a preliminary study. In future studies, we will conduct these experiments with the identified “favorable” species, such as *Bifidobacterium*, and the predictive model constructed in our study could be attempted to verify by other cohort in the future.

Despite differences among our study and other similar studies regarding study design, experimental methods and measurements, or subject population dynamics, our results, together with those of other studies, supported that the gut microbiota might be used to noninvasively predict the efficacy and irAEs of ICI-based therapy. In addition, the combination of favorable microbiota with PD-1 blockade–based therapy is promising.

## Data availability statement

The datasets presented in this study can be found in online repositories. The names of the repository/repositories and accession number(s) can be found below: https://www.ncbi.nlm.nih.gov/bioproject/PRJNA847263.

## Ethics statement

The studies involving human participants were reviewed and approved by Institutional ethics committee of Chinese PLA General Hospital. The patients/participants provided their written informed consent to participate in this study.

## Author contributions

ZW and YH conceived and designed the study. ZW and LL collected samples and completed the laboratory experiments. ZW and DH collected the data. ZW, SZ, and ZH analyzed the data. ZW, SZ, and LL drafted the manuscript. The other authors revised the manuscript. All authors have reviewed and approved the manuscript.

## Funding

This study was supported by the Major Research plan of the National Health Commission (No.GWJJ2021100304) and by the Military Health Special Research Project (No.20BJZ37).

## Conflict of interest

The authors declare that the research was conducted in the absence of any commercial or financial relationships that could be construed as a potential conflict of interest.

The reviewer GS declared a shared parent affiliation with the author(s) to the handling editor at the time of review.

## Publisher’s note

All claims expressed in this article are solely those of the authors and do not necessarily represent those of their affiliated organizations, or those of the publisher, the editors and the reviewers. Any product that may be evaluated in this article, or claim that may be made by its manufacturer, is not guaranteed or endorsed by the publisher.

## References

[1] Havel JJ Chowell D Chan TA . The evolving landscape of biomarkers for checkpoint inhibitor immunotherapy. Nat Rev Cancer (2019) 19(3):133–50. doi: 10.1038/s41568-019-0116-x PMC6705396 30755690

[2] Zhou J Mahoney KM Giobbie-Hurder A Zhao F Lee S Liao X . Soluble PD-L1 as a biomarker in malignant melanoma treated with checkpoint blockade. Cancer Immunol Res (2017) 5(6):480–92. doi: 10.1158/2326-6066.CIR-16-0329 PMC5642913 28522460

[3] Garon EB Rizvi NA Hui R Leighl N Balmanoukian AS Eder JP . Pembrolizumab for the treatment of non-small-cell lung cancer. N Engl J Med (2015) 372(21):2018–28. doi: 10.1056/NEJMoa1501824 25891174

[4] Hellmann MD Ciuleanu TE Pluzanski A Lee JS Otterson GA Audigier-Valette C . Nivolumab plus ipilimumab in lung cancer with a high tumor mutational burden. New Engl J Med (2018) 378(22):2093–104. doi: 10.1056/NEJMoa1801946 PMC7193684 29658845

[5] Domingo E Camps C Kaisaki PJ Parsons MJ Mouradov D Pentony MM . Mutation burden and other molecular markers of prognosis in colorectal cancer treated with curative intent: results from the QUASAR 2 clinical trial and an Australian community-based series. Lancet Gastroenterol Hepatol (2018) 3(9):635–43. doi: 10.1016/S2468-1253(18)30117-1 PMC6088509 30042065

[6] Kim ST Cristescu R Bass AJ Kim KM Odegaard JI Kim K . Comprehensive molecular characterization of clinical responses to PD-1 inhibition in metastatic gastric cancer. Nat Med (2018) 24(9):1449–58. doi: 10.1038/s41591-018-0101-z 30013197

[7] Le DT Durham JN Smith KN Wang H Bartlett BR Aulakh LK . Mismatch repair deficiency predicts response of solid tumors to PD-1 blockade. Science (2017) 357(6349):409–13. doi: 10.1126/science.aan6733 PMC5576142 28596308

[8] Vetizou M Pitt JM Daillere R Lepage P Waldschmitt N Flament C . Anticancer immunotherapy by CTLA-4 blockade relies on the gut microbiota. Science (2015) 350(6264):1079–84. doi: 10.1126/science.aad1329 PMC4721659 26541610

[9] Sivan A Corrales L Hubert N Williams JB Aquino-Michaels K Earley ZM . Commensal bifidobacterium promotes antitumor immunity and facilitates anti-PD-L1 efficacy. Science (2015) 350(6264):1084–9. doi: 10.1126/science.aac4255 PMC4873287 26541606

[10] Mager LF Burkhard R Pett N Cooke NCA Brown K Ramay H . Microbiome-derived inosine modulates response to checkpoint inhibitor immunotherapy. Science (2020) 369(6510):1481–9. doi: 10.1126/science.abc3421 32792462

[11] Gopalakrishnan V Spencer CN Nezi L Reuben A Andrews MC Karpinets TV . Gut microbiome modulates response to anti-PD-1 immunotherapy in melanoma patients. Science (2018) 359(6371):97–103. doi: 10.1126/science.aan4236 29097493 PMC5827966

[12] Peng Z Cheng S Kou Y Wang Z Jin R Hu H . The gut microbiome is associated with clinical response to anti-PD-1/PD-L1 immunotherapy in gastrointestinal cancer. Cancer Immunol Res (2020) 8(10):1251–61. doi: 10.1158/2326-6066.CIR-19-1014 32855157

[13] Hakozaki T Richard C Elkrief A Hosomi Y Benlaifaoui M Mimpen I . The gut microbiome associates with immune checkpoint inhibition outcomes in patients with advanced non-small cell lung cancer. Cancer Immunol Res (2020) 8(10):1243–50. doi: 10.1158/2326-6066.CIR-20-0196 32847937

[14] Routy B Le Chatelier E Derosa L Duong CPM Alou MT Daillere R . Gut microbiome influences efficacy of PD-1-based immunotherapy against epithelial tumors. Science (2018) 359(6371):91–7. doi: 10.1126/science.aan3706 29097494

[15] Derosa L Routy B Thomas AM Iebba V Zalcman G Friard S . Intestinal akkermansia muciniphila predicts clinical response to PD-1 blockade in patients with advanced non-small-cell lung cancer. Nat Med (2022) 28(2):315–24. doi: 10.1038/s41591-021-01655-5 PMC9330544 35115705

[16] Iida N Dzutsev A Stewart CA Smith L Bouladoux N Weingarten RA . Commensal bacteria control cancer response to therapy by modulating the tumor microenvironment. Science (2013) 342(6161):967–70. doi: 10.1126/science.1240527 PMC6709532 24264989

[17] Viaud S Saccheri F Mignot G Yamazaki T Daillere R Hannani D . The intestinal microbiota modulates the anticancer immune effects of cyclophosphamide. Science (2013) 342(6161):971–6. doi: 10.1126/science.1240537 PMC4048947 24264990

[18] Fang C Zhong H Lin Y Chen B Han M Ren H . Assessment of the cPAS-based BGISEQ-500 platform for metagenomic sequencing. Gigascience (2018) 7(3):1–8. doi: 10.1093/gigascience/gix133 PMC5848809 29293960

[19] Zheng Y Wang T Tu X Huang Y Zhang H Tan D . Gut microbiome affects the response to anti-PD-1 immunotherapy in patients with hepatocellular carcinoma. J Immunother Cancer (2019) 7(1):193. doi: 10.1186/s40425-019-0650-9 31337439 PMC6651993

[20] Human Microbiome Project C . Structure, function and diversity of the healthy human microbiome. Nature (2012) 486(7402):207–14. doi: 10.1038/nature11234 PMC3564958 22699609

[21] Andrews MC Duong CPM Gopalakrishnan V Iebba V Chen WS Derosa L . Gut microbiota signatures are associated with toxicity to combined CTLA-4 and PD-1 blockade. Nat Med (2021) 27(8):1432–41. doi: 10.1038/s41591-021-01406-6 PMC11107795 34239137

[22] McCulloch JA Davar D Rodrigues RR Badger JH Fang JR Cole AM . Intestinal microbiota signatures of clinical response and immune-related adverse events in melanoma patients treated with anti-PD-1. Nat Med (2022) 28(3):545–56. doi: 10.1038/s41591-022-01698-2 PMC10246505 35228752

[23] Akk abundance alters survival in patients with NSCLC on ICIs. Cancer Discovery (2022) 12(4):OF8. doi: 10.1158/2159-8290.CD-RW2022-027 35149527

[24] Zhang C Wang J Sun Z Cao Y Mu Z Ji X . Commensal microbiota contributes to predicting the response to immune checkpoint inhibitors in non-small-cell lung cancer patients. Cancer Sci (2021) 112(8):3005–17. doi: 10.1111/cas.14979 PMC8353904 34028936

[25] Matson V Fessler J Bao R Chongsuwat T Zha Y Alegre ML . The commensal microbiome is associated with anti-PD-1 efficacy in metastatic melanoma patients. Science (2018) 359(6371):104–8. doi: 10.1126/science.aao3290 PMC6707353 29302014

[26] Frankel AE Coughlin LA Kim J Froehlich TW Xie Y Frenkel EP . Metagenomic shotgun sequencing and unbiased metabolomic profiling identify specific human gut microbiota and metabolites associated with immune checkpoint therapy efficacy in melanoma patients. Neoplasia (2017) 19(10):848–55. doi: 10.1016/j.neo.2017.08.004 PMC5602478 28923537

[27] Derosa L Routy B Fidelle M Iebba V Alla L Pasolli E . Gut bacteria composition drives primary resistance to cancer immunotherapy in renal cell carcinoma patients. Eur Urol (2020) 78: 195–206. doi: 10.1016/j.eururo.2020.04.044 32376136

[28] Kwak SH Cho YM Noh GM Om AS . Cancer preventive potential of kimchi lactic acid bacteria (Weissella cibaria, lactobacillus plantarum). J Cancer Prev (2014) 19(4):253–8. doi: 10.15430/JCP.2014.19.4.253 PMC4285955 25574459

[29] Zhou Z Chen J Yao H Hu H . Fusobacterium and colorectal cancer. Front Oncol (2018) 8:371. doi: 10.3389/fonc.2018.00371 30374420 PMC6196248

[30] Mima K Nishihara R Qian ZR Cao Y Sukawa Y Nowak JA . Fusobacterium nucleatum in colorectal carcinoma tissue and patient prognosis. Gut (2016) 65(12):1973–80. doi: 10.1136/gutjnl-2015-310101 PMC4769120 26311717

[31] Andrews MC Reuben A Gopalakrishnan V Wargo JA . Concepts collide: Genomic, immune, and microbial influences on the tumor microenvironment and response to cancer therapy. Front Immunol (2018) 9:946. doi: 10.3389/fimmu.2018.00946 29780391 PMC5945998

[32] Wang Z Zhao J Wang G Zhang F Zhang Z Zhang F . Comutations in DNA damage response pathways serve as potential biomarkers for immune checkpoint blockade. Cancer Res (2018) 78(22):6486–96. doi: 10.1158/0008-5472.CAN-18-1814 30171052

[33] Paulos CM Wrzesinski C Kaiser A Hinrichs CS Chieppa M Cassard L . Microbial translocation augments the function of adoptively transferred self/tumor-specific CD8+ T cells *via* TLR4 signaling. J Clin Invest (2007) 117(8):2197–204. doi: 10.1172/JCI32205 PMC1924500 17657310

[34] Yin P Liu X Mansfield AS Harrington SM Li Y Yan Y . CpG-induced antitumor immunity requires IL-12 in expansion of effector cells and down-regulation of PD-1. Oncotarget (2016) 7(43):70223–31. doi: 10.18632/oncotarget.11833 PMC5342548 27602959

