# Supplementary material for: The gut microbiota modulates responses to anti–PD-1 and chemotherapy combination therapy and related adverse events in patients with advanced solid tumors
Source: Front Oncol. 2022 Oct 25;12:887383. doi: 10.3389/fonc.2022.887383 (PMC9641019; doi:10.3389/fonc.2022.887383)
Supplement: Supplementary file 1 [file DataSheet_1.pdf]

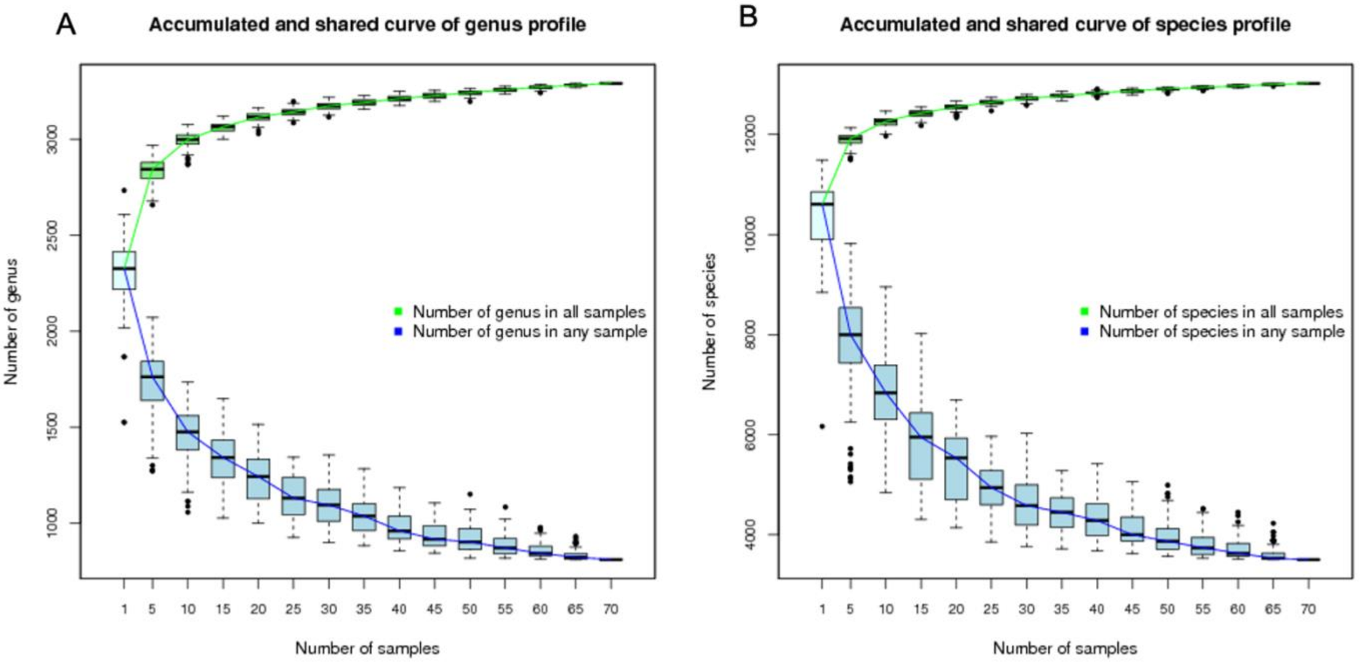

eFigure 1. Accumulated and shared curves of **(A)** genus profile and **(B)** species profile.

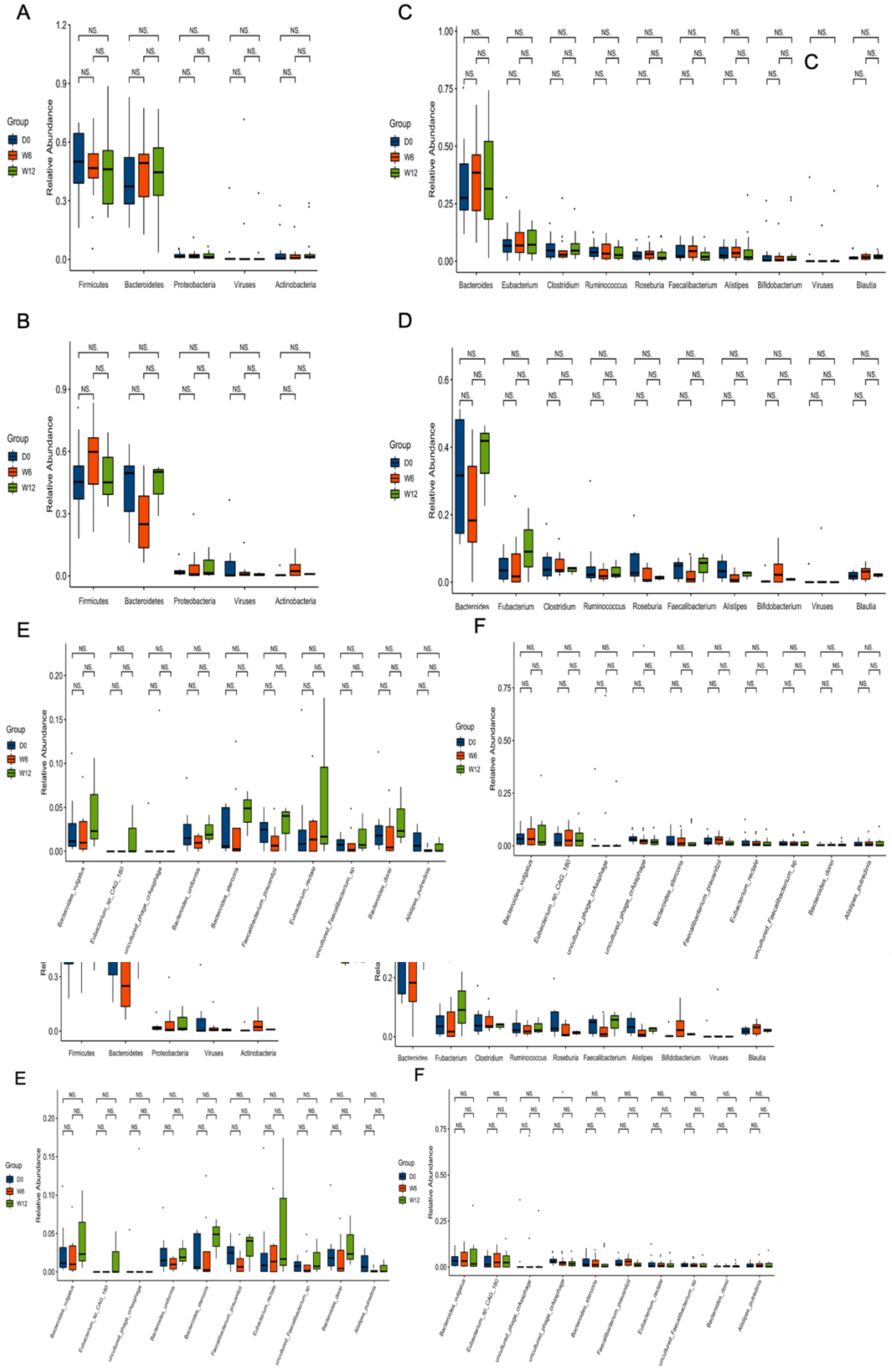

eFigure 2. Dynamic comparison of the dominant gut microbiota longitudinally. **(A) and (B)** Dominant phyla change in R and NR groups; **(C) and (D)** Dominant genera change in R and NR groups; **(E) and (F)** Dominant species change in R and NR groups.

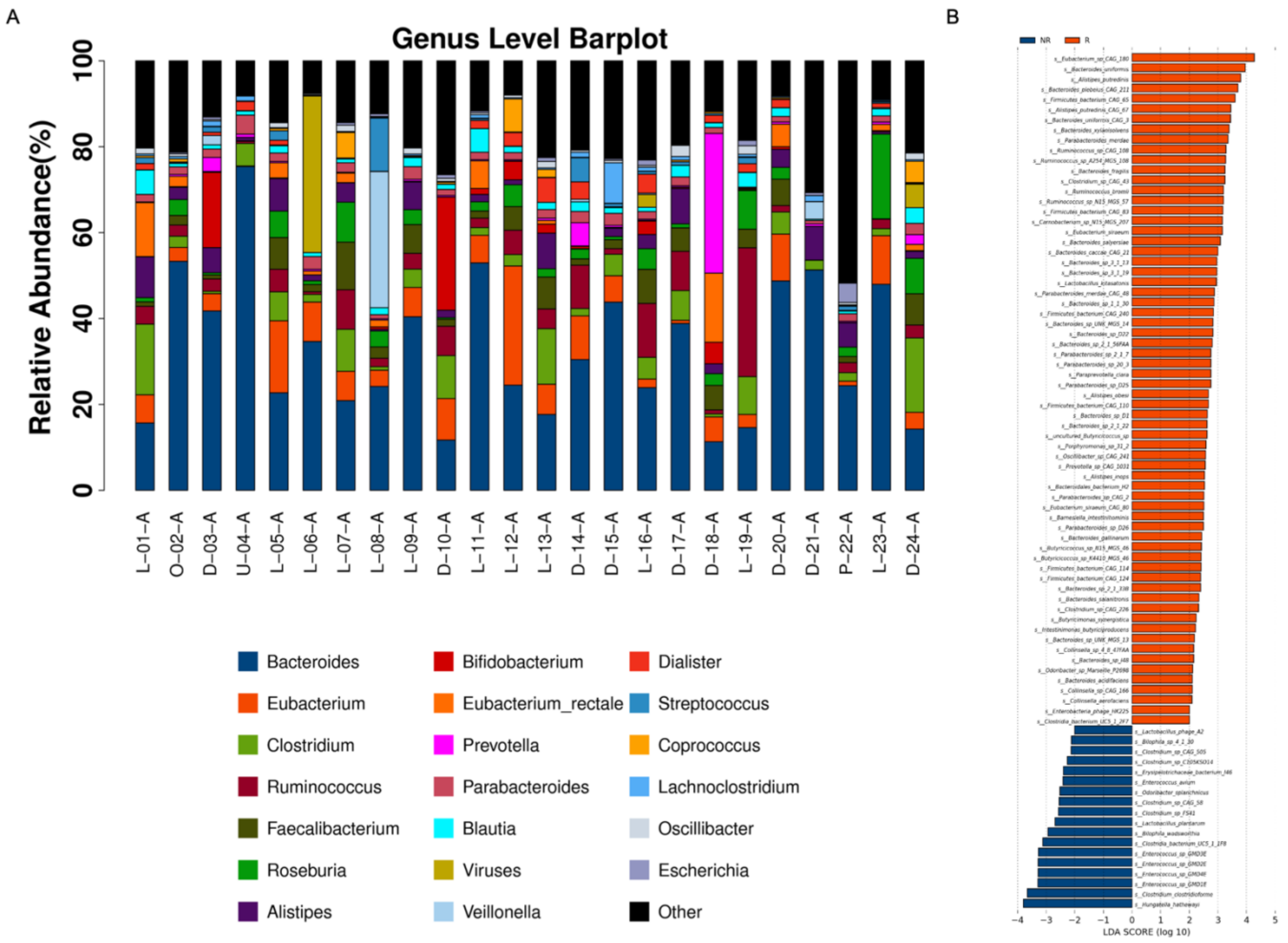

eFigure 3. (A) Distribution of dominant genera of 24 patients at baseline; **(B)** The relative abundance of differential species at 6 weeks, as identified by LEfSe analysis (Kruskal-Wallis sum-rank test, P<0.05)

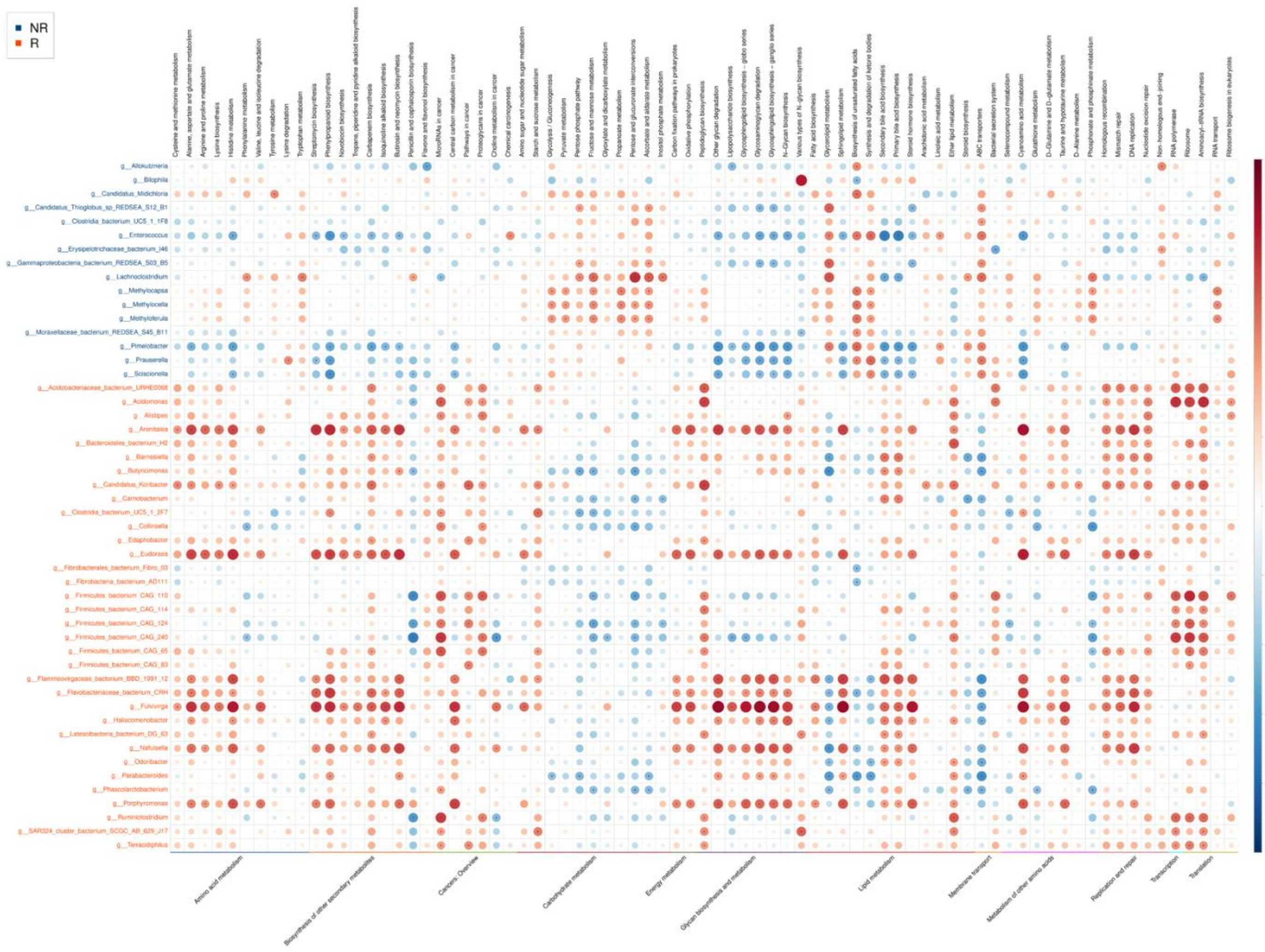

eFigure 4 The correlation analysis of metabolic pathways and differential genera at 6 weeks between R and NR groups (Spearman correlations with rho > 0.5, P < 0.01 were shown as squares in circles).

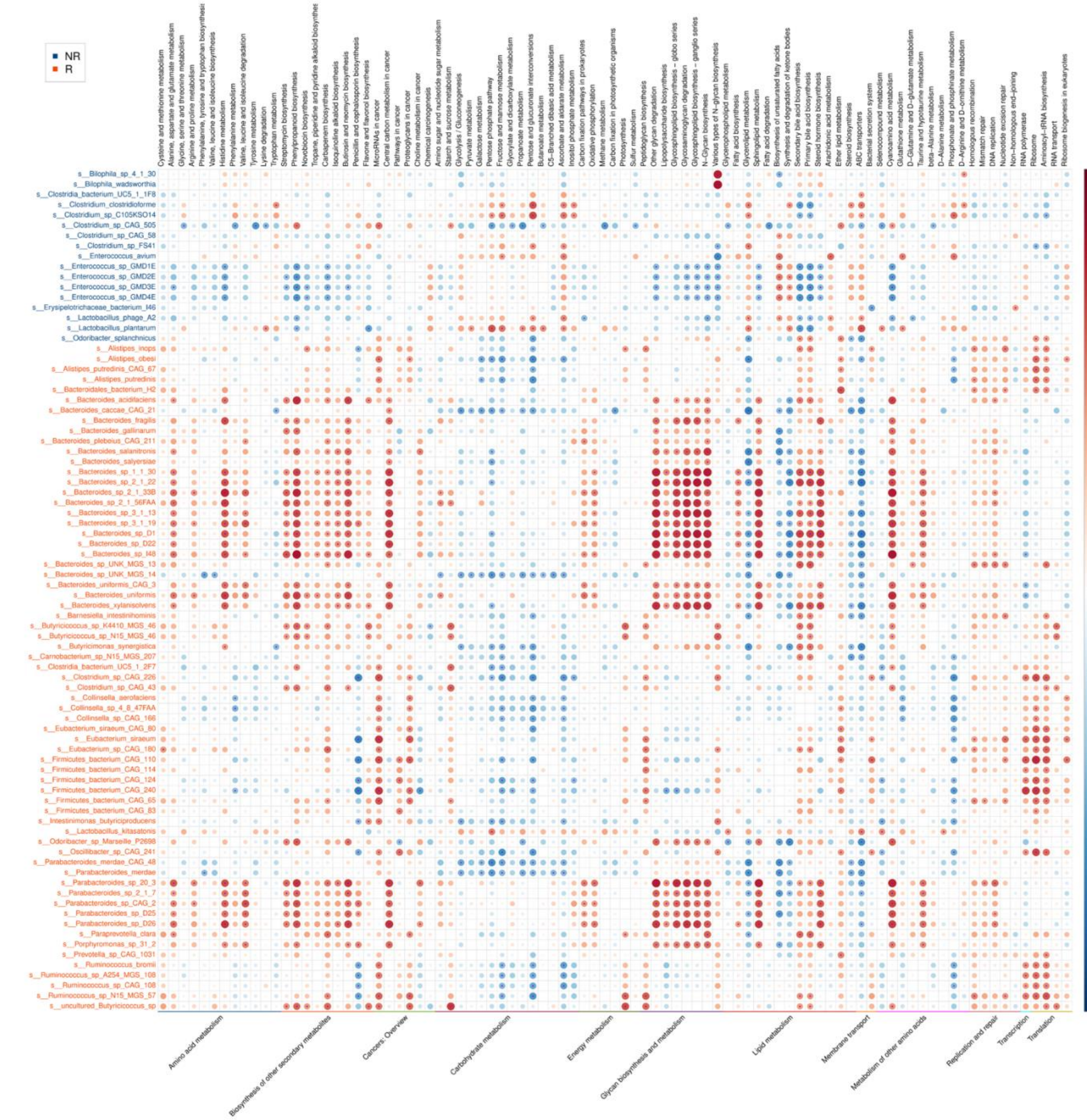

eFigure 5 The correlation analysis of metabolic pathways and differential species at 6 weeks between R and NR groups.

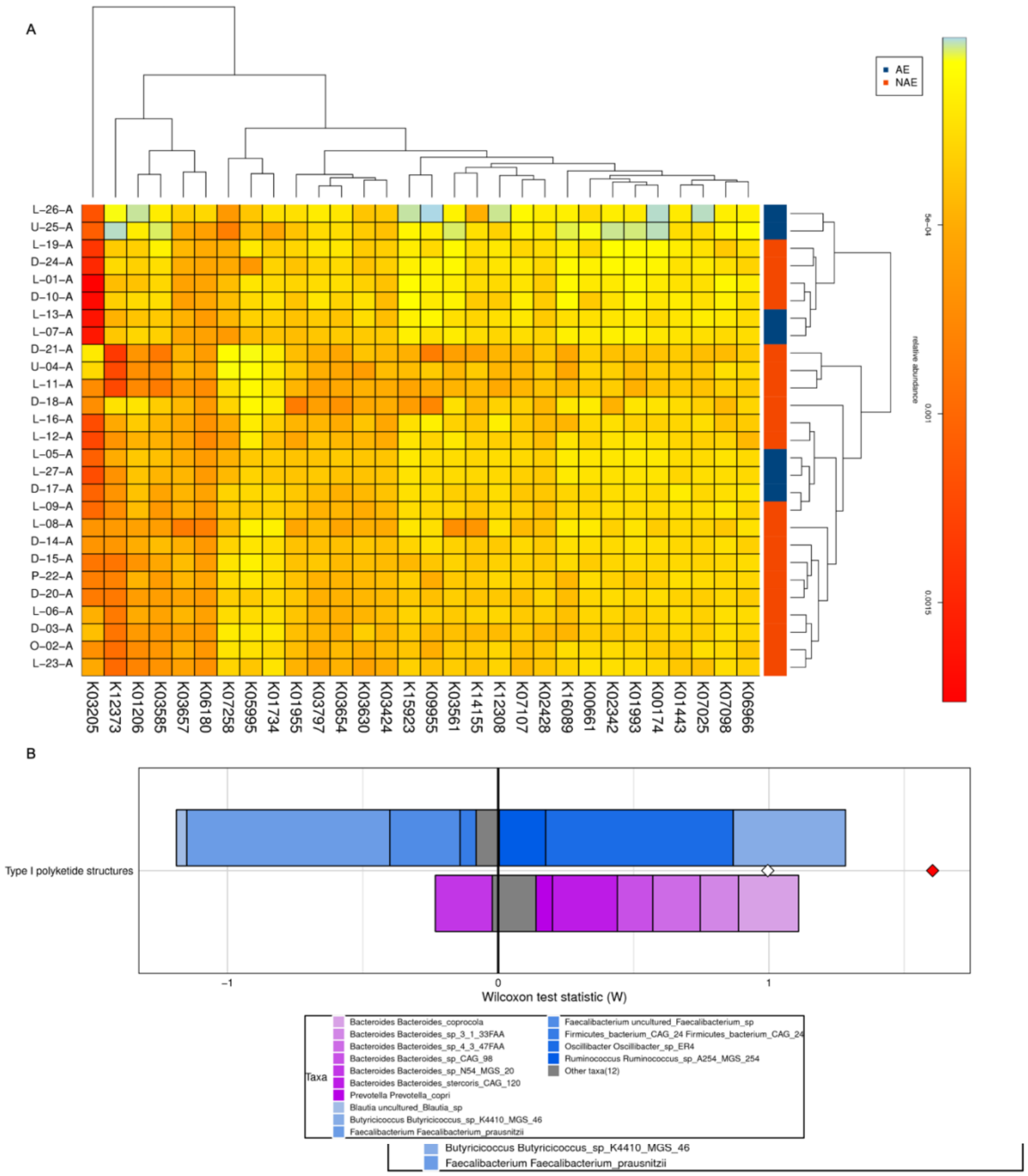

eFigure 6 (A) The heatmap of baseline samples and differential KOs based on AE and evaluated by LEfse analysis. **(B)** Fishtaco analysis of type I polyketide biosynthesis and differential general.

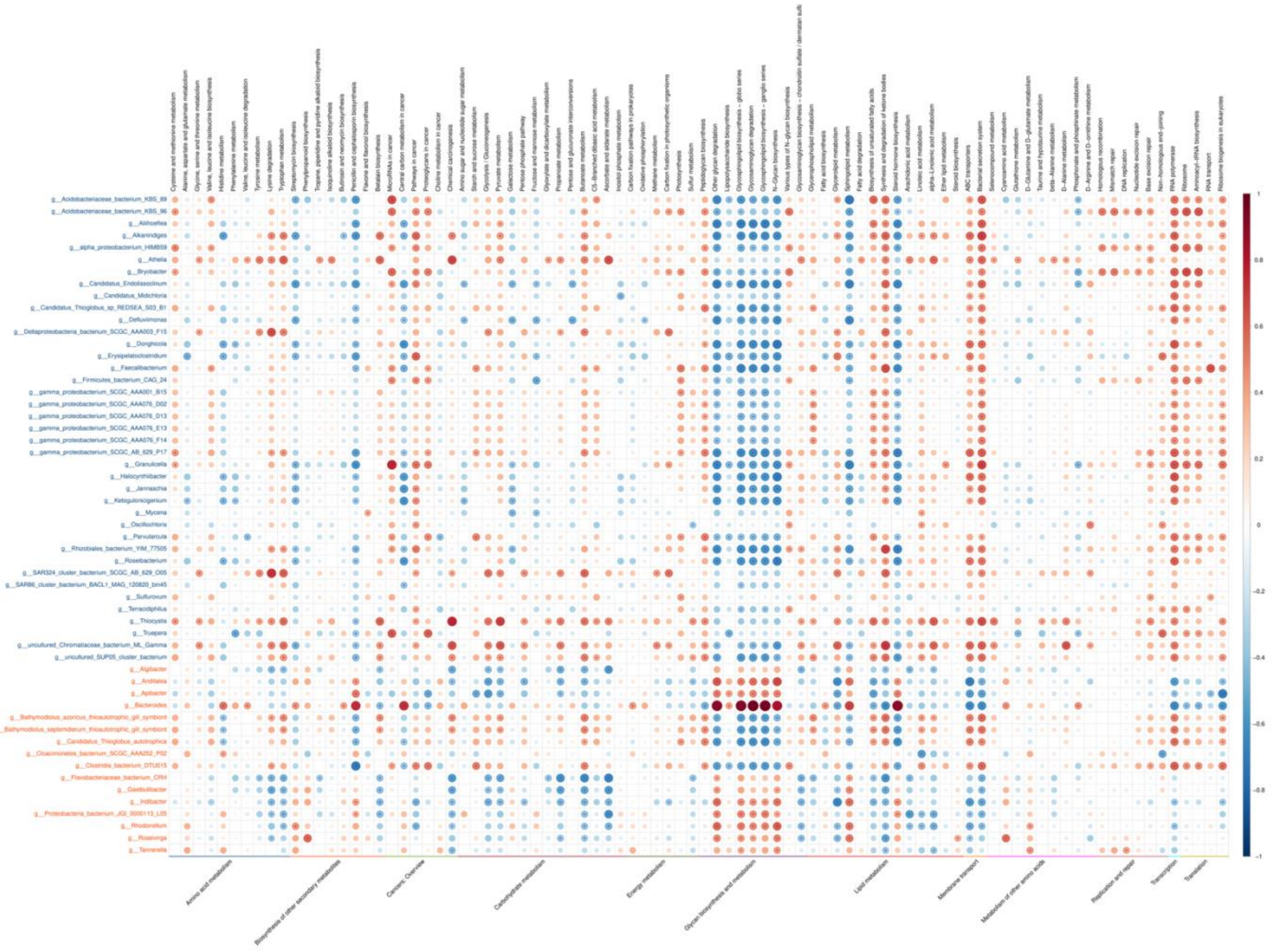

eFigure 7 The correlation analysis of metabolic pathways and differential genera between AE and NAE groups.

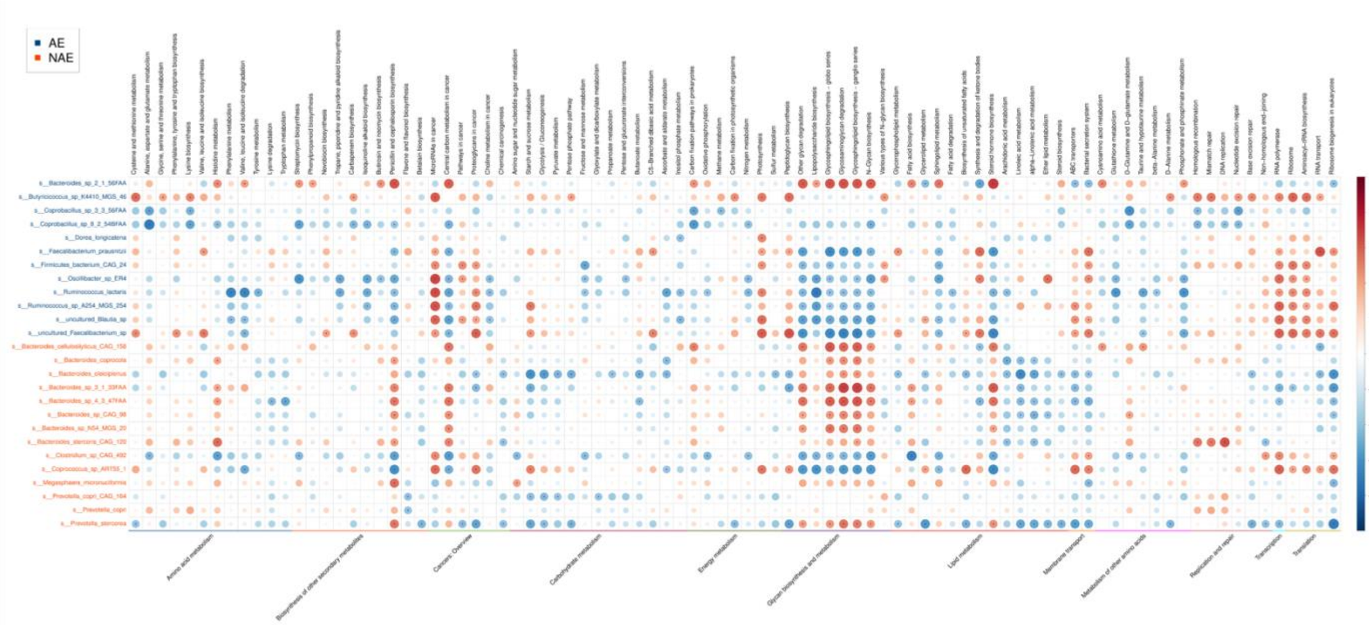

eFigure 8 The correlation analysis of metabolic pathways and differential species between AE and NAE groups.
