# Supplementary material for: The gut microbiota modulates responses to anti–PD-1 and chemotherapy combination therapy and related adverse events in patients with advanced solid tumors
Source: Front Oncol. 2022 Oct 25;12:887383. doi: 10.3389/fonc.2022.887383 (PMC9641019; doi:10.3389/fonc.2022.887383)
Supplement: Supplementary file 2 [file Table_1.docx]

**eTable 1. Raw data of 70 cases of cancer patients’ microbiota with fecal samples.**

| Name | Read length (bp) | Insert size (bp) | Raw reads (#) | Raw bases (bp) | Q20(%) | Q30(%) | GC(%) |
| --- | --- | --- | --- | --- | --- | --- | --- |
| L-01-A | 150 | 371 | 81,718,998 | 12,257,849,700 | 99.18 | 90.75 | 47 |
| L-01-B | 150 | 342 | 122,343,756 | 18,351,563,400 | 98.52 | 89.71 | 47 |
| L-01-C | 150 | 352 | 105,350,316 | 15,802,547,400 | 98.6 | 88.67 | 49 |
| O-02-A | 150 | 344 | 103,391,172 | 15,508,675,800 | 98.41 | 89.05 | 45 |
| O-02-B | 150 | 343 | 101,574,980 | 15,236,247,000 | 99.39 | 92.81 | 46 |
| O-02-C | 150 | 344 | 72,502,032 | 10,875,304,800 | 99.12 | 92.11 | 45 |
| D-03-A | 150 | 342 | 104,186,182 | 15,627,927,300 | 98.9 | 91.19 | 46 |
| D-03-B | 150 | 336 | 92,410,004 | 13,861,500,600 | 99.2 | 92.17 | 45 |
| D-03-C | 150 | 339 | 101,615,938 | 15,242,390,700 | 99.04 | 91.1 | 47 |
| U-04-A | 150 | 353 | 77,415,744 | 11,612,361,600 | 99.19 | 91.76 | 44 |
| U-04-B | 150 | 347 | 88,606,790 | 13,291,018,500 | 99.44 | 93.22 | 44 |
| U-04-C | 150 | 344 | 97,471,038 | 14,620,655,700 | 99.28 | 92.8 | 44 |
| L-05-A | 150 | 347 | 79,327,972 | 11,899,195,800 | 98.83 | 90.38 | 46 |
| L-05-B | 150 | 336 | 91,463,626 | 13,719,543,900 | 98.97 | 91.44 | 45 |
| L-05-C | 150 | 343 | 78,918,454 | 11,837,768,100 | 98.94 | 91.43 | 45 |
| L-06-A | 150 | 340 | 80,427,500 | 12,064,125,000 | 99.29 | 92.55 | 44 |
| L-06-B | 150 | 465 | 123,790,700 | 18,568,605,000 | 98.34 | 86.97 | 42 |
| L-06-C | 150 | 454 | 131,888,864 | 19,783,329,600 | 98.15 | 85.45 | 47 |
| L-07-A | 150 | 395 | 114,120,618 | 17,118,092,700 | 98.52 | 88.69 | 48 |
| L-07-B | 150 | 493 | 118,386,652 | 17,757,997,800 | 97.66 | 86.91 | 47 |
| L-07-C | 150 | 419 | 96,236,044 | 14,435,406,600 | 98.78 | 88.39 | 48 |
| L-08-A | 150 | 383 | 91,124,134 | 13,668,620,100 | 99.04 | 91.38 | 43 |
| L-08-B | 150 | 454 | 116,923,766 | 17,538,564,900 | 97.98 | 83.95 | 47 |
| L-08-C | 150 | 382 | 83,310,112 | 12,496,516,800 | 98.98 | 90.03 | 47 |
| L-09-A | 150 | 408 | 99,557,204 | 14,933,580,600 | 98.13 | 86.8 | 47 |
| L-09-B | 150 | 423 | 122,366,684 | 18,355,002,600 | 97.8 | 85.59 | 48 |
| L-09-C | 150 | 392 | 93,891,662 | 14,083,749,300 | 98.59 | 88.84 | 46 |
| D-10-A | 150 | 419 | 107,683,512 | 16,152,526,800 | 98.04 | 85.68 | 52 |
| D-10-B | 150 | 382 | 79,128,612 | 11,869,291,800 | 98.51 | 88.3 | 49 |
| D-10-C | 150 | 445 | 103,320,266 | 15,498,039,900 | 98.1 | 85.21 | 52 |
| L-11-A | 150 | 334 | 80,980,626 | 12,147,093,900 | 98.6 | 91.13 | 45 |
| L-11-B | 150 | 432 | 110,426,296 | 16,563,944,400 | 98.34 | 87.59 | 47 |
| L-11-C | 150 | 428 | 86,719,356 | 13,007,903,400 | 98.99 | 89.4 | 44 |
| L-12-A | 150 | 418 | 97,042,878 | 14,556,431,700 | 98.82 | 88.11 | 47 |
| L-12-B | 150 | 440 | 83,698,020 | 12,554,703,000 | 98.92 | 88.26 | 47 |
| L-12-C | 150 | 430 | 96,471,660 | 14,470,749,000 | 98.65 | 87.33 | 46 |
| L-13-A | 150 | 451 | 114,679,228 | 17,201,884,200 | 97.87 | 83.35 | 51 |
| L-13-B | 150 | 433 | 109,398,016 | 16,409,702,400 | 98.34 | 85.53 | 49 |
| L-13-C | 150 | 335 | 88,623,644 | 13,293,546,600 | 99.08 | 92.03 | 47 |
| D-14-A | 150 | 361 | 89,428,216 | 13,414,232,400 | 98.82 | 91.08 | 46 |
| D-14-B | 150 | 424 | 94,376,502 | 14,156,475,300 | 98.76 | 87.19 | 45 |
| D-14-C | 150 | 337 | 105,806,816 | 15,871,022,400 | 97.55 | 84.72 | 46 |
| D-15-A | 150 | 428 | 93,855,922 | 14,078,388,300 | 98.31 | 88.57 | 45 |
| D-15-B | 150 | 392 | 99,321,932 | 14,898,289,800 | 98.18 | 87.75 | 48 |
| D-15-C | 150 | 488 | 85,199,532 | 12,779,929,800 | 99.22 | 92.78 | 43 |
| L-16-A | 150 | 399 | 127,318,466 | 19,097,769,900 | 98.51 | 87.39 | 49 |
| L-16-B | 150 | 406 | 122,522,754 | 18,378,413,100 | 97.17 | 83.98 | 48 |
| L-16-C | 150 | 411 | 107,949,120 | 16,192,368,000 | 97.64 | 84.79 | 49 |
| D-17-A | 150 | 352 | 95,741,258 | 14,361,188,700 | 99.01 | 91.18 | 47 |
| D-17-B | 150 | 383 | 99,033,986 | 14,855,097,900 | 98.94 | 90.65 | 47 |
| D-18-A | 150 | 437 | 107,941,544 | 16,191,231,600 | 98.51 | 87.57 | 45 |
| D-18-B | 150 | 408 | 97,871,626 | 14,680,743,900 | 98.34 | 88.62 | 47 |
| L-19-A | 150 | 441 | 100,917,226 | 15,137,583,900 | 98.29 | 87.18 | 45 |
| L-19-B | 150 | 413 | 89,065,976 | 13,359,896,400 | 98.9 | 90.13 | 45 |
| D-20-A | 150 | 406 | 124,559,906 | 18,683,985,900 | 98.37 | 87.77 | 44 |
| D-20-B | 150 | 380 | 103,121,488 | 15,468,223,200 | 99.07 | 92.09 | 43 |
| D-20-C | 150 | 401 | 84,556,074 | 12,683,411,100 | 98.74 | 90.17 | 45 |
| D-21-A | 150 | 409 | 85,612,376 | 12,841,856,400 | 98.53 | 89.22 | 45 |
| D-21-B | 150 | 428 | 97,668,556 | 14,650,283,400 | 98.55 | 88.21 | 49 |
| D-21-C | 150 | 416 | 130,984,222 | 19,647,633,300 | 97.99 | 86.74 | 46 |
| P-22-A | 150 | 442 | 102,522,602 | 15,378,390,300 | 97.67 | 83.81 | 48 |
| P-22-B | 150 | 426 | 104,402,376 | 15,660,356,400 | 97.78 | 85.33 | 50 |
| L-23-A | 150 | 438 | 112,341,596 | 16,851,239,400 | 98.23 | 86.91 | 42 |
| L-23-B | 150 | 350 | 90,089,410 | 13,513,411,500 | 97.93 | 85.23 | 44 |
| L-23-C | 150 | 393 | 102,519,562 | 15,377,934,300 | 99.05 | 90.22 | 43 |
| D-24-A | 150 | 411 | 112,725,398 | 16,908,809,700 | 98.23 | 85.25 | 47 |
| D-24-B | 150 | 426 | 121,099,342 | 18,164,901,300 | 98.28 | 84.9 | 45 |
| U-25-A | 150 | 376 | 77,502,550 | 11,625,382,500 | 98.73 | 88.48 | 45 |
| L-26-A | 150 | 374 | 83,760,778 | 12,564,116,700 | 98.62 | 88.47 | 51 |
| L-27-A | 150 | 400 | 131,338,220 | 19,700,733,000 | 98 | 85.36 | 47 |

**eTable 2. Co-abundance genes (CAG) analysis between R and NR groups.**

| CAG ID | Number of genes(#) | Taxonomy | Level | Number of genes  on DataBase(#) |
| --- | --- | --- | --- | --- |
| 997 | 1614 | Bacteroidetes | phylum | 1519 |
| 1063 | 2079 | Clostridiales | order | 2003 |
| 42 | 2694 | Bacteroidales | order | 2689 |
| 10659 | 1939 | Erysipelotrichaceae | family | 1939 |
| 1421 | 1039 | Enterobacteriaceae | family | 1028 |
| 1621 | 2207 | Lachnospiraceae | family | 2148 |
| 93 | 1658 | Lachnospiraceae | family | 1653 |
| 1199 | 1901 | Alistipes | genus | 1887 |
| 137 | 1372 | Klebsiella | genus | 1347 |
| 15 | 2847 | Bacteroides | genus | 2835 |
| 241 | 1815 | Bacteroides | genus | 1808 |
| 3166 | 1322 | Klebsiella | genus | 1321 |
| 3172 | 739 | Klebsiella | genus | 736 |
| 3448 | 1056 | Klebsiella | genus | 965 |
| 6684 | 2408 | Bilophila | genus | 2377 |
| 677 | 1907 | Bacteroides | genus | 1899 |
| 73 | 1416 | Bifidobacterium | genus | 1415 |
| 860 | 3529 | Bacteroides | genus | 3518 |
| 890 | 1500 | Bacteroides | genus | 1495 |
| 1045 | 804 | Lactobacillus_salivarius | species | 795 |
| 1211 | 865 | Bacteroides_ovatus | species | 793 |
| 133 | 2676 | Bacteroides_caccae | species | 2667 |
| 14 | 2887 | Bacteroides_fragilis | species | 2771 |
| 182 | 1752 | Eubacterium_siraeum | species | 1659 |
| 2270 | 1455 | Bacteroides_finegoldii | species | 1405 |
| 245 | 2213 | Parabacteroides_johnsonii | species | 2150 |
| 2586 | 997 | Eubacterium_rectale | species | 991 |
| 3430 | 821 | Streptococcus_parasanguinis | species | 807 |
| 4388 | 1048 | Streptococcus_thermophilus | species | 993 |
| 5065 | 2390 | Parabacteroides_merdae | species | 2387 |
| 5312 | 2139 | Bacteroides_eggerthii | species | 2123 |
| 6876 | 1572 | Roseburia_intestinalis | species | 1549 |
| 930 | 1926 | Bacteroides_ovatus | species | 1898 |
| 13198 | 1056 | Anaerotruncus_colihominis_DSM_17241 | strain | 1041 |
| 13351 | 1440 | Anaerotruncus_colihominis_DSM_17241 | strain | 1435 |
| 135 | 1739 | Alistipes_putredinis_DSM_17216 | strain | 1697 |
| 1507 | 1619 | Eubacterium_ventriosum_ATCC_27560 | strain | 1491 |
| 155 | 1516 | Faecalibacterium_prausnitzii_L2-6 | strain | 1464 |
| 1598 | 2386 | Clostridium_leptum_DSM_753 | strain | 2148 |
| 1727 | 2165 | Odoribacter_splanchnicus_DSM_20712 | strain | 2061 |
| 2123 | 1402 | Faecalibacterium_prausnitzii_A2_165 | strain | 1360 |
| 2170 | 1257 | Clostridium_hathewayi_DSM_13479 | strain | 1142 |
| 2315 | 1483 | Faecalibacterium_cf__prausnitzii_KLE1255 | strain | 1391 |
| 2433 | 1062 | Roseburia_hominis_A2_183 | strain | 976 |
| 2485 | 1263 | Ruminococcus_obeum_ATCC_29174 | strain | 1201 |
| 250 | 4209 | Lachnospiraceae_bacterium_3_1_57FAA_CT1 | strain | 3883 |
| 2513 | 1414 | Ruminococcus_bromii_L2-63 | strain | 1391 |
| 29 | 1962 | Bacteroides_stercoris_ATCC_43183 | strain | 1927 |
| 2952 | 1297 | Roseburia_inulinivorans_DSM_16841 | strain | 1258 |
| 3027 | 1562 | Coprococcus_comes_ATCC_27758 | strain | 1449 |
| 334 | 1778 | Alistipes_shahii_WAL_8301 | strain | 1737 |
| 3453 | 1339 | Sutterella_wadsworthensis_3_1_45B | strain | 1219 |
| 3889 | 1880 | Subdoligranulum_4_3_54A2FAA | strain | 1832 |
| 3896 | 1664 | Bacteroides_coprocola_DSM_17136 | strain | 1552 |
| 4752 | 714 | Bacteroides_nordii_CL02T12C05 | strain | 703 |
| 5161 | 1214 | Barnesiella_intestinihominis_YIT_11860 | strain | 1188 |
| 52 | 5450 | Clostridium_bolteae_ATCC_BAA_613 | strain | 4956 |
| 5810 | 1327 | Parabacteroides_goldsteinii_CL02T12C30 | strain | 1234 |
| 6511 | 1317 | Ruminococcus_5_1_39BFAA | strain | 1294 |
| 6578 | 1105 | butyrate-producing_bacterium_SS3/4 | strain | 1028 |
| 6720 | 922 | Roseburia_hominis_A2_183 | strain | 883 |
| 7356 | 1230 | Bilophila_wadsworthia_3_1_6 | strain | 1119 |
| 8478 | 2415 | Bacteroides_intestinalis_DSM_17393 | strain | 2266 |
| 8624 | 2734 | Bacteroides_salyersiae_CL02T12C01 | strain | 2719 |
| 949 | 1377 | Dialister_invisus_DSM_15470 | strain | 1327 |

**eTable 3. Different KOs with significant differences between R and NR groups.**

| Taxonname | LDA | P values | Enrichment group |
| --- | --- | --- | --- |
| K10112 | 2.97045419 | 0.03733642 | NR |
| K02032 | 2.65645633 | 0.01996445 | NR |
| K02033 | 3.12003648 | 0.00145086 | NR |
| K02034 | 3.05895069 | 0.01011233 | NR |
| K02035 | 3.05941892 | 0.00219965 | NR |
| K03455 | 2.71061202 | 0.04329747 | R |
| K03523 | 2.87024685 | 0.00400014 | NR |
| K10439 | 3.10103102 | 0.00484876 | NR |
| K00175 | 2.91057533 | 0.01996445 | R |
| K00174 | 2.94269193 | 0.01430588 | R |
| K02768 | 2.78631055 | 0.00845842 | NR |
| K02027 | 3.20798483 | 0.00484876 | NR |
| K02026 | 3.43013164 | 0.00845842 | NR |
| K02025 | 3.42343128 | 0.00328861 | NR |
| K03518 | 2.86132227 | 0.00400014 | NR |
| K03088 | 3.98996636 | 0.01430588 | R |
| K06966 | 2.97525095 | 0.02346498 | R |
| K09808 | 2.83627838 | 0.01692871 | R |
| K04069 | 3.12363845 | 0.02748634 | NR |
| K08218 | 2.76161729 | 0.03733642 | R |
| K02050 | 2.9199278 | 0.00328861 | NR |
| K02051 | 2.83247114 | 0.04329747 | NR |
| K07085 | 3.00357359 | 0.01204828 | R |
| K10907 | 2.82996146 | 0.01011233 | NR |
| K07406 | 2.52344214 | 0.00178957 | NR |
| K07407 | 3.10159824 | 0.03733642 | R |
| K01791 | 2.94451285 | 0.00845842 | R |
| K02863 | 2.97918352 | 0.01430588 | R |
| K02049 | 2.87053846 | 0.00075702 | NR |
| K03797 | 3.12210285 | 0.01430588 | R |
| K07090 | 2.9130444 | 0.03733642 | NR |
| K01077 | 2.79646529 | 0.01011233 | R |
| K00812 | 2.90999531 | 0.01204828 | R |
| K08303 | 3.17038774 | 0.00328861 | R |
| K02526 | 2.44699336 | 0.03733642 | NR |
| K03786 | 2.79537741 | 0.00178957 | R |
| K01869 | 2.90320112 | 0.00094364 | R |
| K05815 | 2.45803177 | 0.00219965 | NR |
| K05814 | 2.53104234 | 0.00400014 | NR |
| K00798 | 2.61396314 | 0.00484876 | R |
| K00791 | 2.93229586 | 0.01011233 | R |
| K01262 | 3.03112852 | 0.00269426 | R |
| K02795 | 2.58090209 | 0.00845842 | NR |
| K02794 | 2.61199787 | 0.00075702 | NR |
| K02796 | 2.54316299 | 0.01430588 | NR |
| K02337 | 3.01571821 | 0.01204828 | R |
| K20263 | 0 | 0.04105089 | NR |
| K10823 | 2.83150709 | 0.00269426 | NR |
| K07056 | 3.04821432 | 0.00094364 | R |
| K00016 | 2.82035033 | 0.00328861 | NR |
| K00876 | 3.06109962 | 0.00178957 | R |
| K03924 | 2.99172548 | 0.01692871 | R |
| K03744 | 2.81671336 | 0.03208873 | R |
| K06158 | 3.03525119 | 0.0058571 | R |
| K01278 | 2.90791585 | 0.02346498 | R |
| K00852 | 2.74524504 | 0.02346498 | NR |
| K01847 | 2.87282496 | 0.00178957 | R |
| K01993 | 2.92669529 | 0.02346498 | R |
| K01190 | 3.57678394 | 0.0058571 | R |
| K01206 | 3.23826532 | 0.01692871 | R |
| K01424 | 3.00479351 | 0.02748634 | R |
| K00640 | 3.01673437 | 0.00269426 | R |
| K05349 | 3.62113134 | 0.01204828 | R |
| K02428 | 3.03196207 | 0.04329747 | R |
| K03773 | 2.7358199 | 0.03733642 | R |
| K12373 | 3.37998777 | 0.03733642 | R |
| K02355 | 3.25740529 | 0.00117214 | R |
| K06180 | 3.28891428 | 0.00845842 | R |
| K10440 | 3.05024345 | 0.00328861 | NR |
| K10441 | 3.03747403 | 0.01011233 | NR |
| K00857 | 2.74487374 | 0.00038266 | R |
| K01187 | 3.2096427 | 0.02748634 | R |
| K01439 | 2.78482506 | 0.0058571 | NR |
| K00926 | 2.75406726 | 0.00178957 | NR |
| K03585 | 3.23830524 | 0.01204828 | R |
| K02429 | 3.02489783 | 0.01204828 | R |
| K07718 | 3.21555008 | 0.00328861 | NR |
| K02759 | 2.46448725 | 0.00400014 | NR |
| K01159 | 2.77505172 | 0.00030268 | R |
| K03718 | 2.81467067 | 0.01996445 | R |
| K03710 | 2.81625978 | 0.03208873 | NR |
| K03484 | 2.71572663 | 0.00060517 | NR |
| K02283 | 2.80628808 | 0.0058571 | NR |
| K00058 | 3.0665188 | 0.01692871 | NR |
| K07010 | 2.51777668 | 0.00845842 | NR |
| K01897 | 3.20753295 | 0.01996445 | R |
| K02760 | 2.54810704 | 0.00145086 | NR |
| K02761 | 2.74039819 | 0.00145086 | NR |
| K03704 | 2.82480449 | 0.02346498 | NR |
| K12340 | 2.75177963 | 0.01692871 | R |
| K00602 | 2.99749988 | 0.00219965 | R |
| K01179 | 2.86595678 | 0.00705073 | R |
| K00951 | 3.05470566 | 0.00705073 | R |
| K02774 | 2.43965721 | 0.00219965 | NR |
| K02773 | 2.46132084 | 0.0058571 | NR |
| K03555 | 2.92624433 | 0.00178957 | R |
| K00639 | 2.76696556 | 0.01692871 | R |
| K07816 | 3.00519522 | 0.03208873 | NR |
| K07720 | 3.19754147 | 0.00400014 | NR |
| K00566 | 3.15655632 | 0.00705073 | R |
| K02014 | 3.23980805 | 0.04329747 | R |
| K02010 | 2.79036092 | 0.03733642 | NR |
| K02122 | 2.6653267 | 0.0058571 | NR |
| K03832 | 2.81349611 | 0.02748634 | R |
| K03614 | 2.88020389 | 0.00400014 | R |
| K01610 | 2.91055451 | 0.03208873 | R |
| K11072 | 2.85215775 | 0.00328861 | R |
| K19271 | 2.87140338 | 0.01204828 | R |
| K05879 | 2.50932708 | 0.02748634 | NR |
| K01284 | 2.90257648 | 0.02346498 | R |
| K02755 | 2.79517838 | 0.00038266 | NR |
| K01572 | 3.08207024 | 0.01011233 | R |
| K02005 | 3.09581191 | 0.01011233 | R |
| K01624 | 3.19903733 | 0.00178957 | NR |
| K06901 | 2.94080295 | 0.00705073 | NR |
| K00761 | 2.94542349 | 0.00030268 | R |
| K18785 | 2.66192005 | 0.01204828 | R |
| K10206 | 3.00653506 | 0.01996445 | R |
| K01960 | 2.66869692 | 0.03208873 | R |
